# Transcriptome-wide comparison of the impact of Atoh1 and miR-183 family on pluripotent stem cells and multipotent otic progenitor cells

**DOI:** 10.1371/journal.pone.0180855

**Published:** 2017-07-07

**Authors:** Michael Ebeid, Prashanth Sripal, Jason Pecka, Kirk W. Beisel, Kelvin Kwan, Garrett A. Soukup

**Affiliations:** 1 Department of Biomedical Sciences, Creighton University, Omaha, Nebraska, United States of America; 2 Department of Cell Biology and Neuroscience, W. M. Keck Center for Collaborative Neuroscience, Rutgers University, Piscataway, New Jersey, United States of America; Texas A&M University, UNITED STATES

## Abstract

Over 5% of the global population suffers from disabling hearing loss caused by multiple factors including aging, noise exposure, genetic predisposition, or use of ototoxic drugs. Sensorineural hearing loss is often caused by the loss of sensory hair cells (HCs) of the inner ear. A barrier to hearing restoration after HC loss is the limited ability of mammalian auditory HCs to spontaneously regenerate. Understanding the molecular mechanisms orchestrating HC development is expected to facilitate cell replacement therapies. Multiple events are known to be essential for proper HC development including the expression of Atoh1 transcription factor and the miR-183 family. We have developed a series of vectors expressing the miR-183 family and/or Atoh1 that was used to transfect two different developmental cell models: pluripotent mouse embryonic stem cells (mESCs) and immortalized multipotent otic progenitor (iMOP) cells representing an advanced developmental stage. Transcriptome profiling of transfected cells show that the impact of Atoh1 is contextually dependent with more HC-specific effects on iMOP cells. miR-183 family expression in combination with Atoh1 not only appears to fine tune gene expression in favor of HC fate, but is also required for the expression of some HC-specific genes. Overall, the work provides novel insight into the combined role of Atoh1 and the miR-183 family during HC development that may ultimately inform strategies to promote HC regeneration or maintenance.

## Introduction

Cochlear hair cells (HCs) within the inner ear are the mechanoreceptor epithelial cells of the auditory system. These cells are vulnerable to damage by different factors including noise, drugs or aging. The inability of mammalian HCs to regenerate after ototoxic damaged leads to sensorineural hearing loss [1]. Hearing loss is a major health concern that affects over 5% of the world’s population, (approximately 360 million people) [2]. Currently, the only clinical treatment for a severe-to-profound sensorineural hearing loss is cochlear implantation, which provides variable outcomes [3]. Current research hopes to provide a basis for HC regeneration or replacement therapies that will restore natural hearing. Such efforts require better understanding of the molecular mechanisms orchestrating HC development and maintenance.

Development of the inner ear requires a precisely timed cascade of molecular events leading to a sequence of cell fate determinations. The molecular mechanisms guiding this process are not fully elucidated, yet some genes are shown to be crucial for the process. One of the key events is the expression of a proneural basic helix-loop-helix (bHLH) transcription factor Atoh1, which is necessary for HC development and is thought to be the earliest determinant of HC fate [4,5]. Furthermore, overexpression of Atoh1 is shown to be contextually sufficient to drive ectopic HC generation [6,7]. Since Atoh1 is crucial for differentiation of other neuronal cell types such as cerebellar granule cells [8] and spinal cord interneurons [9], as well as non-neuronal cell types such as intestinal secretory cells [10], it is believed that the cellular context is an important determinant of Atoh1 function. Previous studies have described some Atoh1 target genes in the cerebellum [11] and the developing spinal cord [12] utilizing chromatin immunoprecipitation and sequencing. Yet only a handful of Atoh1 target genes have been validated for HCs [13]. To date, the molecular mechanisms underlying the role of Atoh1 in HC differentiation and the contextual prerequisite for such a role are not well understood.

Another level of gene regulation during development is achieved by small non-coding RNAs termed microRNAs (miRNAs). These RNAs post-transcriptionally silence complementary target mRNAs [14]. A number of miRNAs are expressed in the mammalian inner ear and may contribute to proper development of the sensory epithelia including the miR-183 family (*miR-183*, *miR-96* and *miR-182*). These miRNAs are coordinately expressed from one primary miRNA transcript [15], and family member sequence and expression are highly conserved in neurosensory organs across phyla [16]. Mutations in *miR-96* seed sequence, the region corresponding to nucleotides 2–8 that interact with target mRNAs, underlie the genetic cause of progressive hearing loss in humans [17]. Previous studies investigated the role of miR-183 family in HC development and maintenance [18–20], yet few target genes have been validated. The molecular mechanisms underlying such role are yet to be identified.

In this work, we investigate the impact of Atoh1 and/or miR-183 family expression on the transcriptomes of two different cell models; pluripotent mouse embryonic stem cells (mESCs) representing a developmentally naive context and mouse multipotent otic progenitor (iMOP) cells as an otic fate-restricted context. Our study provides novel insight into the contextually-dependent role of Atoh1 and miR-183 family in HC development.

## Materials and methods

### Expression vectors

The miRNASelect pEGP-miR cloning and expression vector (Cell Biolabs) was used as a backbone for construction expression vectors. Three miR-183 family members (*miR-183*, *miR-96*, and *miR-182*) were inserted into the human beta globin intron, and the GFP-Puro cassette was replaced with a bicistronic cassette encoding Myc-tagged murine Atoh1 and a tandem dimer Tomato red fluorescence protein (RFP). Atoh1 and RFP coding sequences were separated by a picornaviral 2A peptide coding sequence that enables production of individual products upon translation. From this parent construct termed p183AT, a series of expression vectors were constructed by deleting the Myc-tagged Atoh1 coding sequence and/or the miRNAs (Table 1). The entire expression cassette of each plasmid was sequence verified to ensure design integrity. Each plasmid vector was also converted and produced as an adenoviral vector (Vector Biolabs).

**Table 1:** Plasmid (p) and adenoviral (Ad) vector nomenclature and content.

Vector Nomenclature	Expressed Components		
	miR-183 family	Atoh1	RFP
pT/Ad-T			+
p183T/Ad-183T	+		+
pAT/Ad-AT		+	+
p183AT/Ad-183AT	+	+	+

### Cell culture and transfection

Mouse embryonic stem cells (mESCs) were obtained from The American Type Culture Collection (ATCC ES-D3 cell line). Cells were cultured on a confluent layer of mitotically arrested mouse embryonic fibroblasts in KnockOut DMEM (Life Technologies) containing 15% stem cell-certified fetal bovine serum (Life Technologies), 1000 U/mL mouse leukemia inhibitory factor (Millipore), 1% penicillin-streptomycin and 1% non-essential amino acids. Mouse immortalized multipotent otic progenitor (iMOP) cells are a monoclonal fate-restricted cell line generated by transient expression of c-Myc in *SOX2*-expressing otic progenitor cells obtained from E11.5 mouse cochlea [21]. iMOP cells were cultured in DMEM/F12 Ham’s media (Life Technologies) containing 25 μg/ml carbenecillin, B27 serum free supplement (Life Technologies) and 20ng/mL recombinant murine FGF-basic (Peprotech). HEK293 cells used for validation of prepared vectors were obtained from ATCC. Cells were cultured in DMEM media (Life Technologies) containing 5% fetal bovine serum, 1% penicillin-streptomycin-amphotericin and 1% non-essential amino acids.

Transfection of mESCs was conducted 24 hours after plating using Lipofectamine 2000 (Life Technologies) according to the manufacturer protocol, and cells were incubated for 48 hours. Jet Prime transfection reagent (Polyplus) was used to transfect iMOP cells according to the manufacturer protocol, and transfected cells were incubated for 48 hours. HEK293 cells at 90% confluency were infected with the adenoviral vectors using a cell:viral particle ratio of 1:10 and analyzed 48 hours post-infection.

### Cell sorting and RNA isolation

Fluorescence activated flow cytometric analysis and sorting of RFP-positive cell was performed using a FACS Aria instrument (BD Biosciences) with a 610/20 nm band-pass filter. Approximate expression efficiency was assessed from 10,000 total cells by determining the percentage of cells that were RFP positive compared to untransfected control. Fluorescence activated cell sorting was performed in the same session and RFP-positive mESC cells were collected in fresh media supplemented with 30% stem cell-certified FBS. iMOP cells were sorted directly into lysis buffer with subsequent RNA extraction.

Total RNA was isolated from RFP-positive cell populations immediately after sorting using the mirVana miRNA Isolation Kit (Life Technologies) according to the manufacturer protocol. RNA integrity was assessed using a Bioanalyzer (Agilent) and total RNA was stored at -80°C.

### Microarray analysis

Microarray analysis was performed using 200 ng of total RNA from each sample. RNA was reverse transcribed using the WT PLUS Reagent Kit (Affymetrix, Santa Clara, CA) according to the manufacturer protocol. Hybridization, washing and staining were performed using protocols and reagents recommended for GeneChip Mouse Gene 2.0 ST Arrays (Affymetrix) and the Affymetrix Gene Chip System. All arrays passed the quality control metrics for hybridization kinetics, background and specificity as outlined by Affymetrix for the 2.0 ST Array. Expression Console software (Affymetrix) was used to normalize data and generate probeset intensity values, and Transcriptome Analysis software (Affymetrix) was used to statistically analyze data using a one-way ANOVA test. Heat maps, hierarchical clustering and Venn diagrams for gene expression analysis were generated using R Bioconductor [22]. The data discussed in this study have been deposited in NCBI's Gene Expression Omnibus and are accessible through GEO Series accession number GSE81667. An analysis of raw data are provided in S1 and S2 Tables.

### Reverse transcription and quantitative PCR analysis (qRT-PCR)

For mRNA detection, the TaqMan Reverse Transcription Kit (Life technologies) was used to reverse-transcribe RNA according to the manufacturer protocol. qPCR was performed using Taqman Fast Advanced Universal PCR Master Mix (Life Technologies), and reactions were analyzed using a 7500 Fast Real-Time PCR Instrument (Applied Biosystems). The complete list of Taqman assay IDs are provided in S3 Table. Data analysis was performed using the comparative C_T_ method, and statistical significance was determined using a two-tailed Student’s t-test (p<0.05). For validation experiments, a distinct RNA samples from that used for microarray analysis was generated to ensure reproducibility of results.

For mature miRNA detection, poly(A) tailing and reverse transcription of total RNA was conducted using the NCode VILO miRNA cDNA Synthesis Kit (Invitrogen) according to the manufacturer protocol. qPCR was performed using the Express Sybr Green ER miRNA qRT-PCR Kit (Invitrogen), and reactions were analyzed using a 7500 Fast Real-Time PCR instrument (Applied Biosystems). Primers used for specific RNA detection included U6 5′-GCAAATTCGTGAAGCGTTCCAT, miR-21 5′-CGGTAGCTTATCAGACTGATGTTGA, miR-183 5′-GGTATGGCACTGGTAGAATTCACT, miR-96 5′-TGGCACTAGCACATTTTTGCT, miR-182 5′-CTTGGCAATGGTACAACTCACA.

### Western blot analysis

Total protein was extracted from cell lysates, and Bradford assays were used to determine protein concentration. Samples were separated on 4–20% Mini-Protean Tgx Precast polyacrylamide gels (Bio-Rad). Gels were transferred to a nitrocellulose membrane using the iBlot 7-Minute Blotting System (Invitrogen). Membranes were blocked overnight using 5% milk protein and subsequently incubated at 4°C with monoclonal mouse anti-Myc primary antibody (ABM) for 24 hours. The blot was washed and incubated for 1 hour with a goat anti-mouse horseradish peroxidase-conjugated secondary antibody (Santa Cruz) diluted 1:4000. Horseradish peroxidase activity was detected using the SuperSignal West Femto Maximum Sensitivity Substrate Kit (Thermo Scientific), and chemiluminescence was detected using a ChemiDoc XRS+ Molecular Imager (Bio-Rad).

## Results

### Vector expression of miR-183 family members and Atoh1 protein

**Fig 1:**
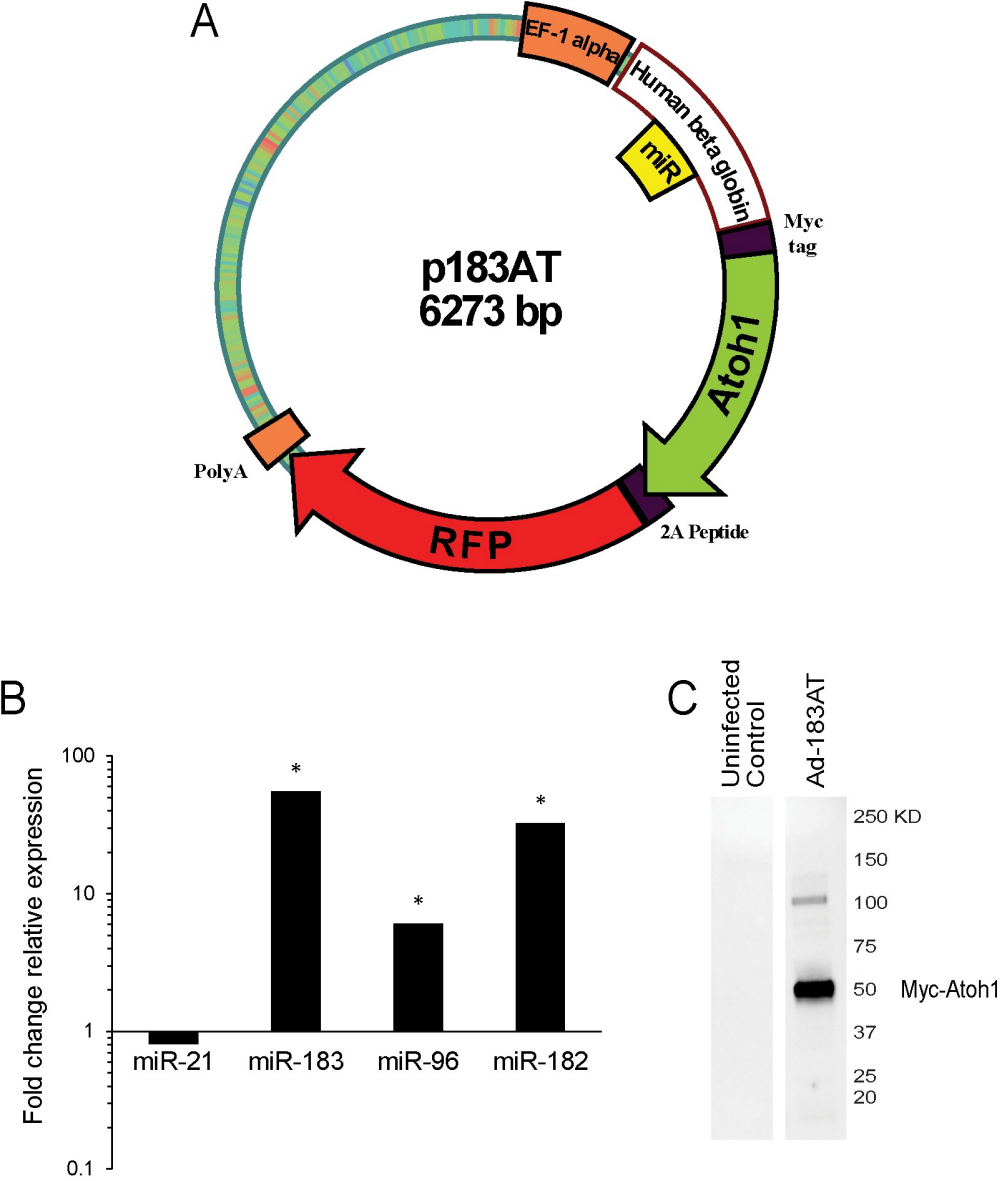
Expression vector design and validation. (A) Schematic diagram of the parent expression vector p183AT for combined production of miR-183 family members (miR), Atoh1 transcription factor, and red fluorescent protein (RFP). The primary transcript is produced from an EF-1 alpha promoter, the miRNAs are processed from a human beta globin intron, and Atoh1 and RFP are separately produced by picornaviral 2A peptide “cleavage”. (B) qRT-PCR validation of miR-183 family member expression in HEK 293 cells. Shown is fold change in expression of miR-21, miR-183, miR-96, and miR-182 in total RNA from Ad-183T infected cells relative to Ad-T. miRNA detection levels were normalized to U6 snRNA. Asterisks indicate statistically significant differences (p<0.05) determined using a two-tailed, type 2 Student’s t-test (n = 3). (C) Western blot analysis of lysates from uninfected control and Ad-183AT infected HEK293 cells showing Myc-tagged Atoh1 detection at the approximate expected size of 43 KDa.

HEK293 cells were used to validate miR-183 family and Atoh1 expression from vectors derived from the parent construct (Fig 1A). qRT-PCR analysis of mature miRNA expression demonstrated a statistically significant increase in miR-183 (55 fold), miR-96 (6 fold) and miR-182 (33 fold) in cells infected with Ad-183T compared to cells infected with the control vector Ad-T (Fig 1B). Conversely, there was no significant change in unrelated miR-21 expression. Western blot analysis using anti-Myc antibody confirmed the presence of Myc-tagged Atoh1 protein in lysates from cells infected with Ad-183AT compared to uninfected control (Fig 1C). A band at the approximate expected size for Myc-tagged Atoh1 (43 KDa) confirms production of individual proteins using the picornaviral 2A peptide sequence, whereas a relatively smaller amount of a higher molecular weight protein likely represents conjugated Atoh1-RFP production that reflects a slight inefficiency in picornaviral 2A peptide “cleavage”.

### mESC and iMOP cell transfection efficiency

**Fig 2:**
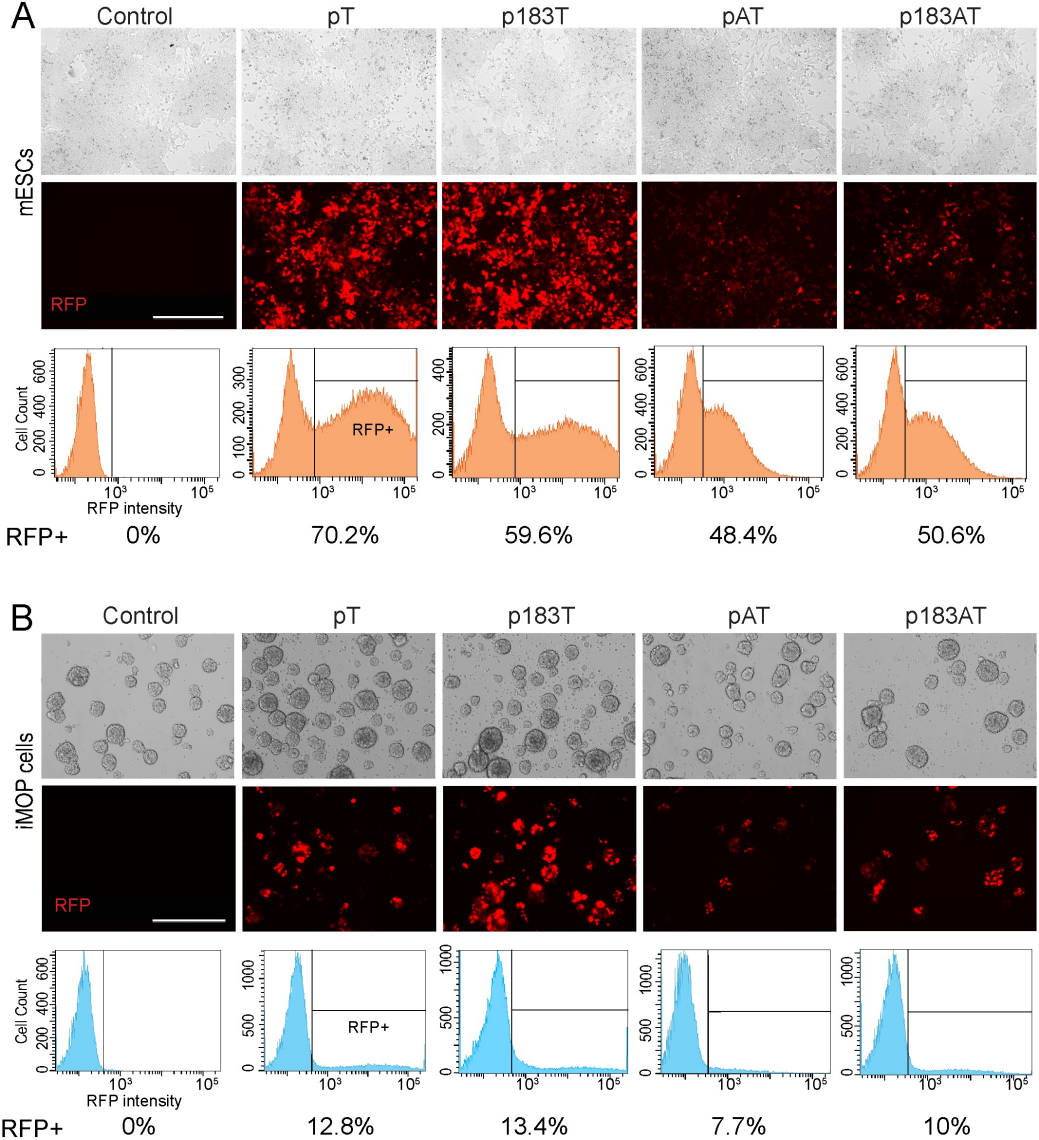
Fluorescence visualization and flow cytometric analysis of mESC and iMOP transfected cells. (A) mESCs at 48 h post-transfection with each of four plasmid vectors. (B) iMOP cells at 48 h post-transfection with each of four plasmid vectors. Bright field and fluorescence images are shown for corresponding fields (bar = 400 μm). Graphs depict representative results from flow cytometric analysis, where the threshold for RFP-positive cells was defined based on analysis of background levels of fluorescence detection in untransfected control cells. The average percentage of RFP-positive cells is indicated (n = 3).

mESCs were not amenable to infection with adenovirus vectors (data not shown). Therefore mESCs and iMOP cells were transfected with each plasmid vectors (pT, pAT, p183T or p183AT) and analyzed 48 h post-transfection. RFP expression was confirmed by fluorescence microscopy and the percent RFP-positive cells was determined using flow cytometric analysis as the average of three different samples for each vector (Fig 2). In mESCs, pT-transfected cells showed the highest percentage of RFP-positive cells with an average transfection efficiency of 70.2% (Fig 2A). In general, iMOP cells exhibited poorer transfection efficiency (Fig 2B), where the percentage of RFP-positive cells was approximately one-fifth that for mESCs (Fig 2B). Additionally, there was an observed decrease in RFP expression from bicistronic vectors producing both Atoh1 and RFP versus monocystronic vectors producing only RFP. Nevertheless, fluorescence-activated cell sorting (FACS) enabled isolation of 2x10^5^ to 1x10^6^ RFP-positive cells from each transfected culture, from which total RNA was extracted for microarray and qRT-PCR analyses.

### Differentially expressed genes in unmanipulated mESCs and iMOP cells

**Fig 3:**
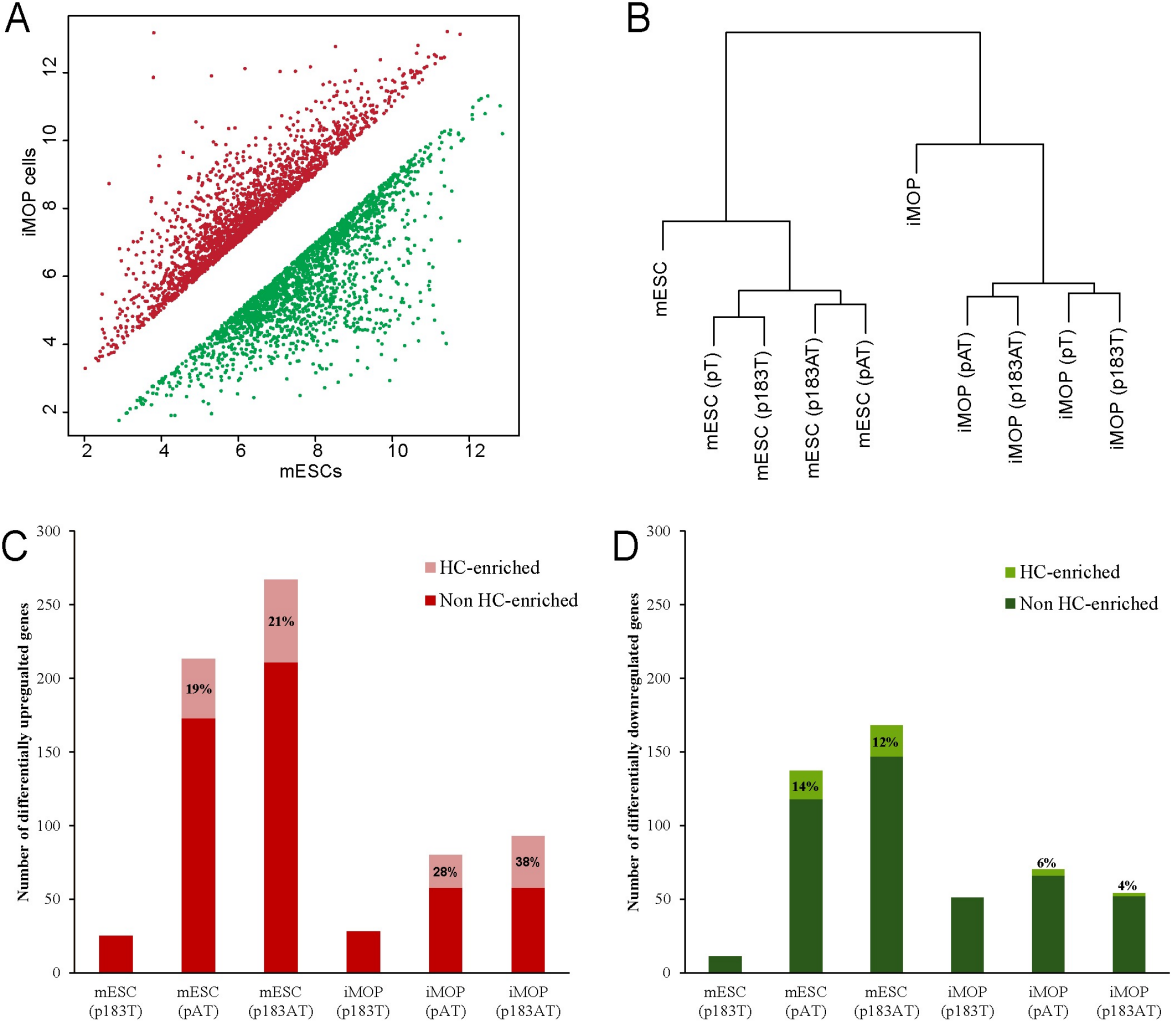
Microarray analysis comparing gene expression of mESCs and iMOP cells. (A) Normalized mean intensity values of differentially expressed genes in untransfected mESC and iMOP cells (n = 3, linear fold change >2 and ANOVA p<0.05). Upregulated genes in iMOP versus mESCs are depicted in red and downregulated genes in green. (B) Hierarchal clustering of gene expression profiles for untransfected mESC and iMOP cells, including profiles for each cell type transfected with indicated expression vector. (C) Number of upregulated genes in transfected mESCs and iMOP cells relative to control pT transfected cells (n = 3, linear fold change ≥1.5 and ANOVA p<0.05). Indicated are upregulated HC-enriched genes (pink) and upregulated non-HC genes (red). (D) Number of downregulated genes in transfected mESCs and iMOP cells relative to control pT transfected cells (n = 3, linear fold change ≥1.5 and ANOVA p<0.05). Indicated are HC-enriched genes (bright green) and non-HC genes (dark green).

Before evaluating the effect of Atoh1 and the miR-183 family on the transcriptomes of cells, we first compared the transcriptomes of untransfected mESCs and iMOP cells. Microarray analysis of global gene expression showed 3623 differentially expressed genes, of which 1694 genes were upregulated and 1929 genes were downregulated in iMOP cells relative to mESCs (n = 3, linear fold change >2 and ANOVA p<0.05) (Fig 3A). The data demonstrate substantially different gene expression profiles for the cell culture models, which can be considered as distinctly different platforms for assessing the roles of Atoh1 and the miR-183 family.

Among genes that showed more than 5-fold upregulation in iMOP cells, there are five genes with known roles in inner ear development: *Dlx5* (26.2 fold), *Pou3f4* (18.12 fold), *Zic1* (11.71 fold), *Insig1* (5.39 fold) and *Eya1* (5.33 fold). No upregulation in expression of either *Atoh1* or the miR-183 family was observed in iMOP cells, indicating that the cell model is derived from cochlear progenitors prior to *Atoh1* expression. Thusly, iMOP cells appear to model a developmental time point representative of a progenitor state before cell cycle exit and differentiation.

### Differentially expressed genes in cells expressing Atoh1 and the miR-183 family

To determine the effects of expressing different combinations of Atoh1 and the miR-183 family in each cell culture model, we determined the number of differentially expressed genes following transfection with each expression vector relative to control pT transfection (n = 3, linear fold change ≥ 1.5 and ANOVA p<0.05). Hierarchical clustering of each transcriptome revealed a clear segregation between cell types (Fig 3B). Differences in transcriptomes were detected between control and miR-183 family-expressing cells as one group, and Atoh1-expressing cells and Atoh1/miR-183 family-expressing cells as another group.

To examine the biological relevance of differentially expressed genes, we compared our results to a P0 cochlear HC transcriptome [13]. We gathered a set of ~3000 genes that showed more than two-fold statistically significant enrichment in nascent HCs versus remaining cochlear cells at P0. In mESCs, Atoh1 expression upregulated 213 genes, 40 of which are enriched in HCs representing 19% of total upregulated genes (Fig 3C). miR-183 family expression upregulated 25 genes, none of which are enriched in HCs. mESCs expressing both miR-183 family and Atoh1 upregulated 267 genes, 56 of which are enriched in HCs (21%). In iMOP cells, Atoh1 expression upregulated 80 genes including 22 enriched in HC (28%). miR-183 family expression upregulated 28 genes, none of which are enriched in HCs. Cells expressing both Atoh1 and miR-183 family exhibited upregulation in 94 genes, 35 of which are enriched in HCs (38%). In general, more genes were upregulated in transfected mESCs, yet the percentage of HC-enriched genes was higher in transfected iMOP cells (Fig 3C). We similarly identified a subset of HC-enriched genes that were downregulated for each transfection condition (Fig 3D). The number of downregulated genes was similarly greater in mESCs except that miR-183 family expression alone exhibited a greater impact on iMOP cells.

### Impact of Atoh1 is dependent on developmental context

**Fig 4:**
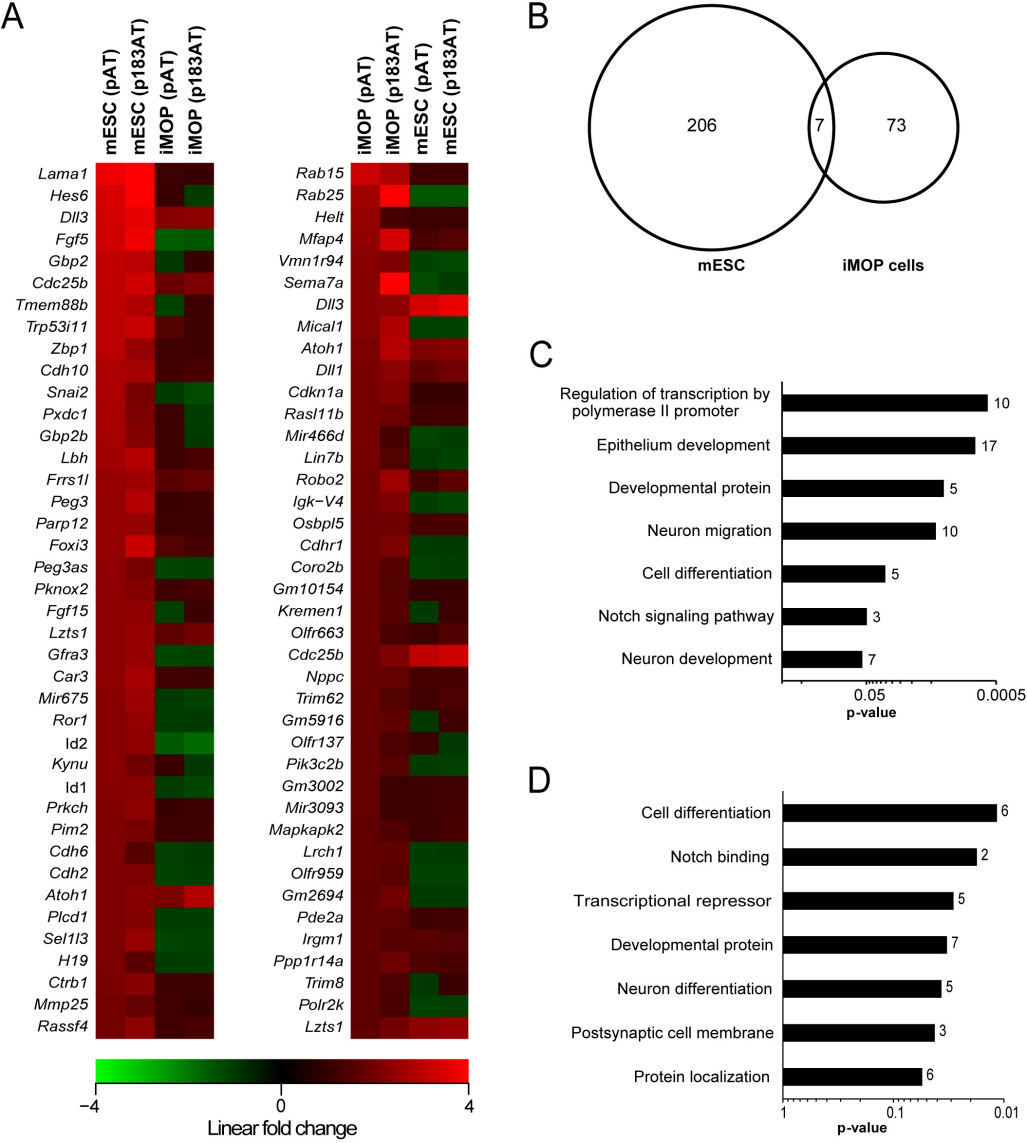
Impact of Atoh1 on mESC versus iMOP cell transcriptomes. (A) Heat maps showing linear fold changes for 40 genes in the transcriptomes of transfected mESC and iMOP cells compared to controls. The genes were selected by comparing Atoh1-expressing cells to control for each cell type (n = 3, linear fold change ≥1.5 and ANOVA p<0.05), and they represent the most upregulated genes in mESCs (left heat map) and iMOP cells (right heat map). Red boxes indicate upregulated genes, green boxes indicate downregulated genes, and black boxes indicate genes with no change in expression. Genes are ordered by greatest fold change in Atoh1-transfected cells (top) to least (bottom). (B) Venn diagram depicting the commonality of differentially upregulated genes among Atoh1-expressing mESC and iMOP cells. (C, D) Graphs showing enriched gene ontological categories in Atoh1-expressing mESCs (C) and iMOP cells (D) with statistical significance (p<0.05). The number of genes within each category is indicated beside each bar.

Comparing the transcriptomes of Atoh1-expressing and control mESCs, 350 genes were differentially expressed; 213 were upregulated and 137 were downregulated (n = 3, linear fold change ≥ 1.5 and ANOVA p<0.05) (Fig 3C and 3D). The ten most upregulated genes were *Lama1*, *Hes6*, *Dll3*, *Fgf5*, *Gbp2*, *Cdc25b*, *Tmem88b*, *Trp53i11*, *Zbp1* and *Cdh10* (Fig 4A). Forty genes out of the 213 were shown to be enriched in P0 cochlear HCs including *Lama1*, *Hes6*, *Dll3*, *Fgf5*, *Tmem88b*, *Lbh*, *Fgf15*, *Gfra3*, *Id2* and *Cdh2*. To assess the impact of Atoh1 on the pluripotency of mESCs, several pluripotency markers were found to be downregulated including *Nanog*, *Nr5a2*, *Prdm14*, *Eras*, *Zfp42*, *Nr0b1*, *Sox15* and *Klf4*.

Comparing the transcriptomes of Atoh1-expressing and control iMOP cells, 150 genes were differentially expressed; 80 genes were upregulated and 70 were downregulated (n = 3, linear fold change ≥ 1.5 and ANOVA p<0.05) (Fig 3C and 3D). The ten most upregulated genes were *Rab15*, *Rab25*, *Helt*, *Mfap4*, *Vmn1r94*, *Sema7a*, *Dll3*, *Mical1*, *Atoh1 and Dll1*(Fig 4A). The number of genes enriched in HCs within this set was 22 including *Rab15*, *Rab25*, *Sema7a*, *Dll3*, *Dll1*, *Lin7b*, *Cdhr1*, *Kremen1*, *Trim62* and *GM2694*.

Comparing the differentially expressed genes in Atoh1-expressing mESCs and Atoh1-expressing iMOP cells, only seven genes were upregulated in both cell types (*Cdc25b*, *Dll1*, *Dll3*, *Dnah7a*, *Frrs1l*, *Irgm1* and *Lzts*) (Fig 4B). Interestingly, three genes showed opposite expression patterns. Both *Fgf5* and *Id2* were upregulated in mESCs and downregulated in iMOP cells, while *Rab25* was upregulated in iMOP cells and downregulated in mESCs.

To identify potential Atoh1 targets, we cross-referenced our data set with Atoh1 target genes from mouse cerebellum [11]. We found that among the 213 upregulated genes in Atoh1-expressing mESCs, 17 genes were identified as Atoh1 targets in cerebellum (*Fgd4*, *Sema6a*, *Hes6*, *Rab30*, *Nxn*, *Vat1l*, *Srrm4*, *Jakmip2*, *Peg3*, *Cdc25b*, *Thsd7a*, *Nnt*, *Rassf4*, *Id2*, *Id1*, *Dll3* and *Baalc*). For iMOP cells, 6 genes among the 80 upregulated genes were identified as Atoh1 targets in cerebellum (*Mfap4*, *Rdh5*, *Sema7a*, *Gadd45g*, *Cdc25b* and *Dll3*)

To investigate the biological processes associated with Atoh1 expression, we utilized the Database for Annotation, Visualization and Integrated Discovery (DAVID) for functional annotation of differentially expressed genes [23,24]. DAVID uses the gene ontology classifications of associated genes with biological processes, and determines which processes are represented in a specific gene list. Gene ontological analyses of Atoh1-upregulated genes in mESCs and iMOP cells revealed a set of functional categories (Fig 4C and 4D) with some common terms such as Notch signaling, developmental proteins, cell differentiation and neuron development. Ontological terms unique to Atoh1-expressing mESCs were cadherin and epithelial development proteins, while Atoh1-expressing iMOP cells showed postsynaptic cell membrane and protein localization terms.

### miR-183 family has greater impact on iMOP cells

**Fig 5:**
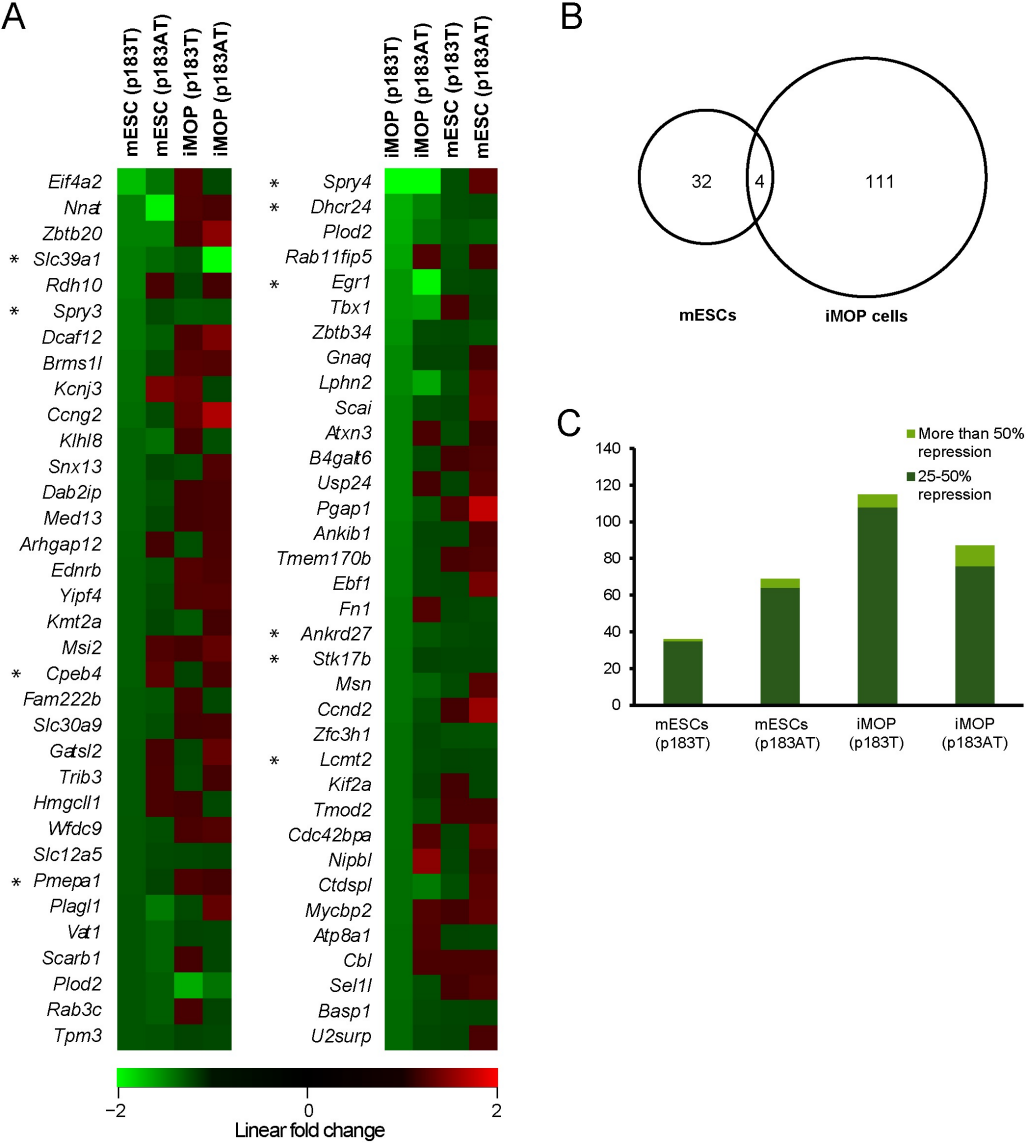
miR-183 family impact on predicted target genes’ expression. (A) Heat maps depicting linear fold changes for 34 genes among miR-183 family predicted target genes in the transcriptomes of transfected mESC and iMOP cells compared to controls. The genes were selected by comparing miR-183 family-expressing cell to control for each cell type (n = 3, linear fold change <-1.25), and they represent the most downregulated genes in mESCs (left heat map) and iMOP cells (right heat map). Red boxes indicate upregulated genes, green boxes indicate downregulated genes, and black boxes indicate genes with no change in expression. Genes are ordered by greatest repression in miR-183 family-transfected cells (top) to least (bottom). Asterisks denote statistically significant differences (p<0.05) using a one-way ANOVA test. (B) Venn diagram depicting the commonality of differentially downregulated miR-183 family predicted target genes among miR-183 family-expressing mESC and iMOP cells. (C) Number of differentially downregulated miR-183 family predicted target genes in transfected mESC and iMOP cells. Bright green bars depict the subset of these genes with more than 50% repression, while dark green bars represent the subset exhibiting 25% to 50% repression.

Transcriptome analysis of miR-183 family-expressing versus control mESCs revealed upregulation of 25 genes and downregulation of 11 genes, while miR-183 family-expressing iMOP cells exhibited upregulation of 28 genes and downregulation of 51 genes (n = 3, linear fold change ≥ 1.5 and ANOVA p<0.05) (Fig 3C and 3D). To assess potential direct effects of the miR-183 family, we utilized the Target Scan Mouse database 7.1 release [25] to identify the predicted target transcripts for all members of this family. We determined a set of ~1600 combined predicted transcripts with conserved sites for the three miRNAs; *miR-183*, *miR-96* and *miR182*. In mESCs expressing the miR-183 family, 36 predicted target genes were repressed more than 25% with only 3 genes showing statistical significance (ANOVA p<0.05) (Fig 5C). The 5 most repressed predicted target genes were *Eif4a2*, *Ninat*, *Zbtb20*, *Slc39a1* and *Rdh10* (Fig 5A). In iMOP cells transfected with the miR-183 family, 115 predicted target genes showed more than 25% repression with 10 genes showing statistical significance (ANOVA p<0.05) (Fig 5C). The 5 most repressed predicted target genes were *Spry4*, *Dhcr24*, *Plod2*, *Rab11fip5*, *Egr1* and *Tbx1* (Fig 5A). Comparing repressed predicted target genes between mESCs and iMOP cells, only four genes were found in common (*Plod2*, *Slc39a1*, *Spry3* and *Kmt2a*) (Fig 5B).

**Fig 6:**
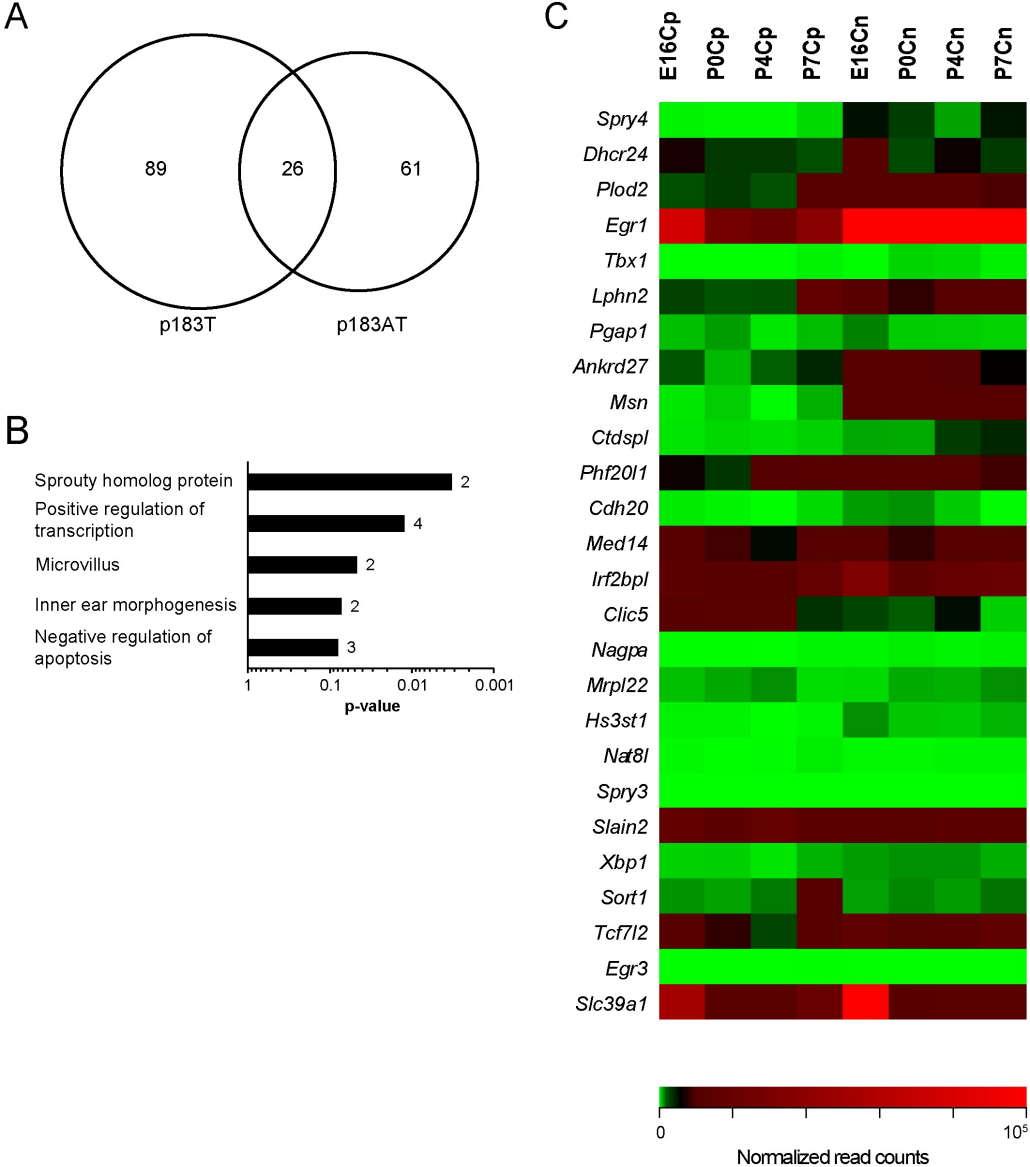
Expression of repressed miR-183 family predicted target genes during inner ear development. (A) Venn diagram depicting the commonality of differentially downregulated miR-183 family predicted target genes among miR-183 family-expressing and the Atoh1/miR-183 family-expressing iMOP cells (n = 3, linear fold change <-1.25). (B) Enriched gene ontological categories among the 26 common repressed predicted target genes with statistical significance (p<0.05). The number of genes within each category is depicted beside each bar. (C) Heat map depicting relative expression levels (normalized read counts) for the 26 common repressed predicted target genes in late prenatal (E16) and early postnatal (P0, P4 and P7) mouse inner ear development of HCs (“p” designation) versus non-HCs (“n” designation) obtained from SHIELD [26]. Red boxes indicate high expression levels, green boxes indicate low expression levels, and black boxes indicate moderate level of expression. Genes are ordered by greatest repression in miR-183 family-expressing iMOP cells (top) to the least (bottom).

Since there were considerably greater numbers of predicted target genes showing repression in transfected iMOP cells, we searched for common repressed predicted target genes in miR-183 family-expressing and Atoh1/miR-183 family-expressing iMOP cells and found 26 genes (Fig 6A). Gene ontological analysis of these targets revealed enrichment in microvillus genes, sprouty proteins, and inner ear morphogenesis terms (Fig 6B). To investigate the biological relevance of these common target genes, we utilized the Shared Harvard Inner Ear Laboratory Database (SHIELD) [26] to understand target gene expression dynamics during normal HC development. Out of the 26 common repressed predicted target genes in miR-183 family-expressing iMOP cells, 14 genes showed consistent repression in cochlear HCs versus non-HCs between E16 and P7 including *Spry4*, *Plod2*, *Egr1*, *Lphn2* and *Ankrd27* (Fig 6C). The data suggest that the miR-183 family contributes to repression of these predicted target genes in vivo.

### Atoh1 and miR-183 family synergism in HC development

**Fig 7:**
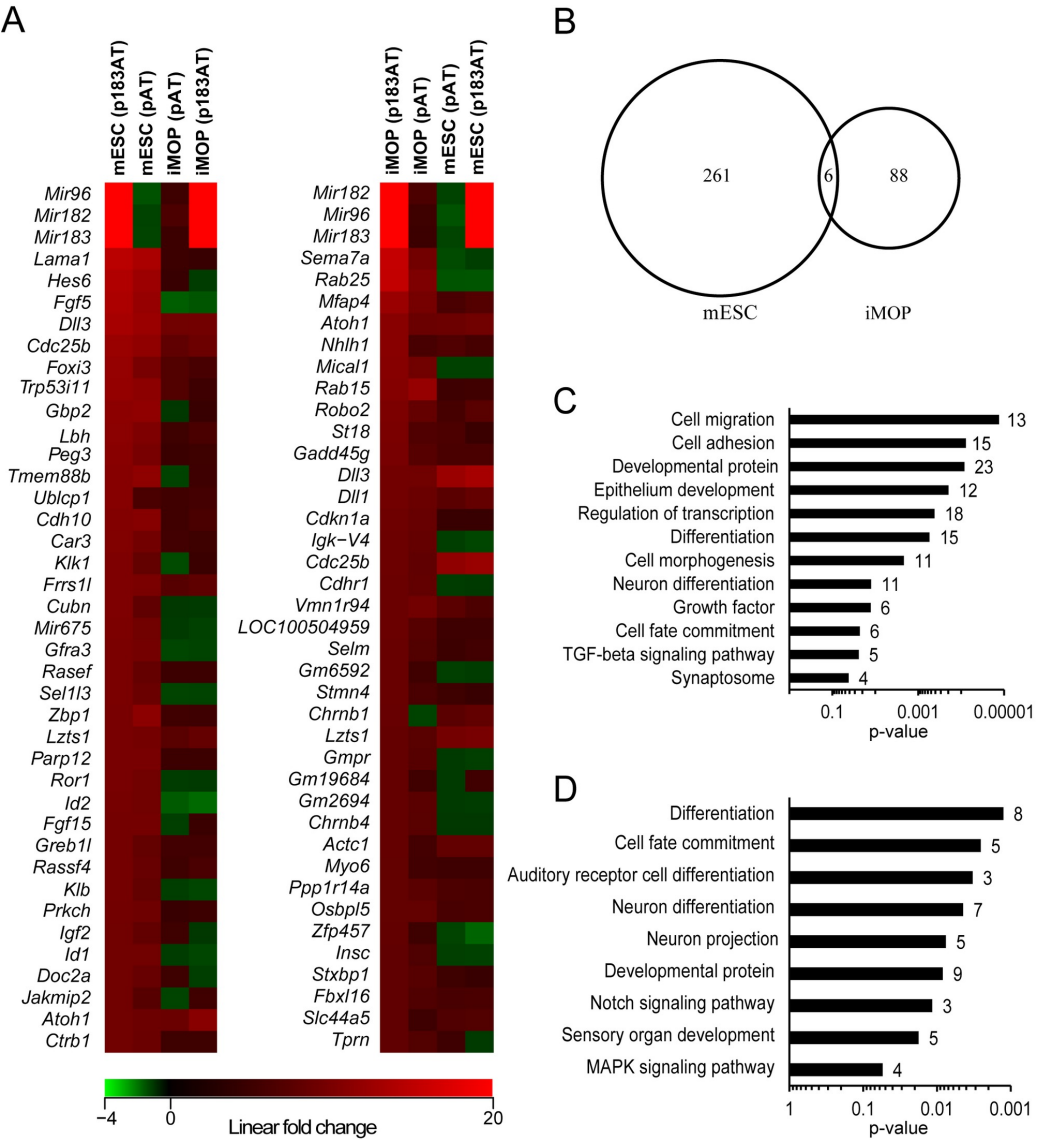
Impact of Atoh1/miR-183 family on mESC and iMOP cell transcriptomes. (A) Heat maps showing linear fold changes for 40 genes in the transcriptomes of transfected mESC and iMOP cells compared to controls. The genes were selected by comparing Atoh1/ miR-183 family-expressing cells to control for each cell type (n = 3, linear fold change ≥1.5 and ANOVA p<0.05), and they represent the most upregulated genes in mESCs (left heat map) and iMOP cells (right heat map). Red boxes indicate upregulated genes, green boxes indicate downregulated genes, and black boxes indicate genes with no change in expression. Genes are ordered by greatest fold change in Atoh1/miR-183 family-expressing cells (top) to least (bottom). (B) Venn diagram showing the commonality of differentially upregulated genes among the Atoh1/miR-183 family-expressing mESC and iMOP cells. (C, D) Graphs show enriched gene ontological categories in Atoh1/miR-183 family-expressing mESCs (C) and iMOP cells (D) with statistical significance (p<0.05). The number of genes within each category is indicated beside each bar.

To examine the effect of combined Atoh1/miR-183 family expression in each cell culture models, the transcriptome of Atoh1/miR-183 family-expressing cells was compared to control in each case. In mESCs, combined expression upregulated 267 genes and downregulated 168 genes, whereas in iMOP cells 94 genes were upregulated and 54 genes were downregulated (n = 3, linear fold change ≥ 1.5 and ANOVA p<0.05) (Fig 3C and 3D). For mESCs, the most upregulated genes were *Mir96*, *Mir182*, *Mir183*, *Lama1*, *Hes6*, *Fgf5*, *Dll3*, *Cdc25b* and *Foxi3*, whereas for iMOP cells the most upregulated genes were: *Mir182*, *Mir96*, *Mir183*, *Sema7a*, *Rab25*, *Mfap4*, *Atoh1*, *Nhlh1*, *Mical1* and *Rab15* (Fig 7A). Comparing upregulated genes in mESCs to the P0 cochlear HC transcriptome [13], we identified 56 genes in common including *Fgf5*, *Lama1*, *Dll3*, *Hes6*, *Gfra3*, *Lbh*, *Sel1l3*, *Tmem88b*, *Id2* and *Rasef*. For iMOP cells, 35 upregulated genes were enriched in cochlear HCs including *Sema7a*, *Rab25*, *Nhlh1*, *Rab15*, *Gadd45g*, *Dll3* and *Dll1*.

Among differentially expressed genes in Atoh1/miR-183 family-expressing mESCs and iMOP cells, there were only 6 upregulated genes in common (*Cdc25b*, *Chrnb1*, *Dll1*, *Dll3*, *Frrs1l* and *Lzts1*) (Fig 7B). Two genes were upregulated in mESCs and downregulated in iMOP cells (*Fgf5* and *Id2*), and one gene was upregulated in iMOP cells and downregulated in mESCs (*Rab25*).

Gene ontological analysis of upregulated genes in Atoh1/miR-183 family-expressing mESCs and iMOP cells showed enrichment of common categories including differentiation, cell fate commitment, developmental proteins and neuron differentiation (Fig 7C and 7D). Unique categories in mESCs included cell migration, epithelial development and TGF-ß signaling, whereas iMOP cells showed enrichment in auditory receptor cell differentiation and MAPK signaling pathway.

**Fig 8:**
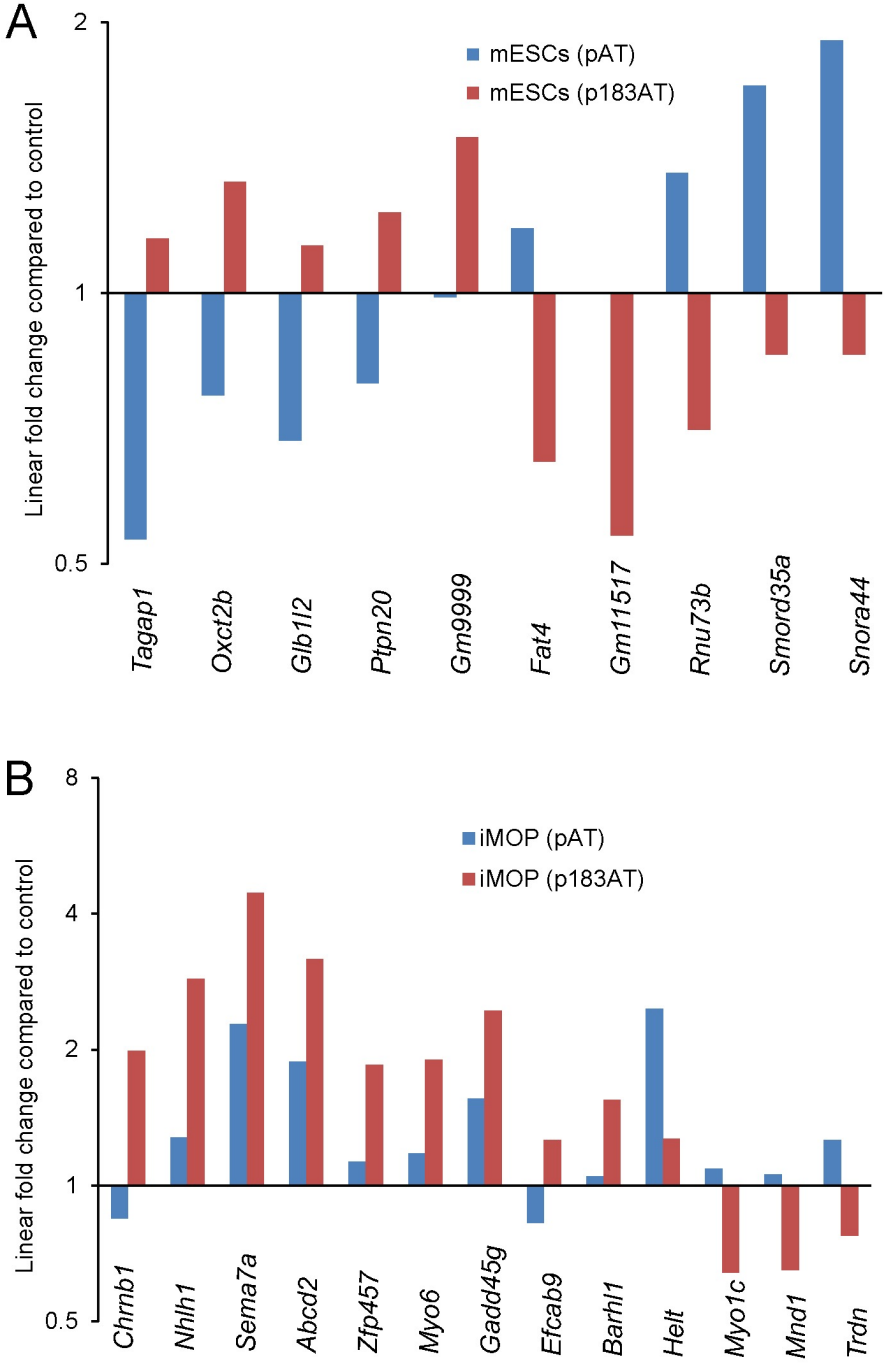
Unique combinational influence of Atoh1 and miR-183 family. (A) Linear fold change in gene expression for Atoh1-expressing mESCs (blue) and Atoh1/miR-183 family-expressing mESCs (red) compared to control. (B) Linear fold change in gene expression for Atoh1-expressing iMOP cells (blue) and Atoh1/miR-183 family-expressing iMOP cells (red) compared to control. Differentially expressed genes for each cell type were selected based on comparison of Atoh1/miR-183 family-expressing cells to Atoh1-expressing cells (n = 3, linear fold change ≥1.5 and ANOVA p<0.06).

To determine how combined Atoh1/miR-183 family expression differs from only Atoh1 expression, we compared the transcriptomes of Atoh1/miR-183 family-expressing cells to Atoh1-expressing cells (linear fold change ≥ 1.5 and ANOVA p<0.06) (Fig 8). In Atoh1/miR-183 family-expressing mESCs, 49 genes were upregulated including 5 enriched in P0 HCs (*Tagap1*, *Oxct2b*, *Glb1l2*, *Ptpn20* and *Gm9999*), and 13 genes were downregulated including 5 that showed lower expression in P0 cochlear HCs relative to supporting cells [13] (*Snora44*, *Snorad35a*, *Rnu73b*, *Gm11517* and *Fat4*). In Atoh1/miR-183 family-expressing iMOP cells, 68 genes were upregulated including 7 genes enriched in HCs (*Chrnb1*, *Nhlh1*, *Sema7a*, *Abcd2*, *Zfp457*, *Myo6* and *Gadd45g*), and 32 genes were downregulated including 4 that showed lower expression in P0 cochlear HCs relative to supporting cells [13] (*Helt*, *Myo1c*, *Mnd1* and *Trdn*) (Fig 8).

### Validation of a subset of differentially expressed genes

**Fig 9:**
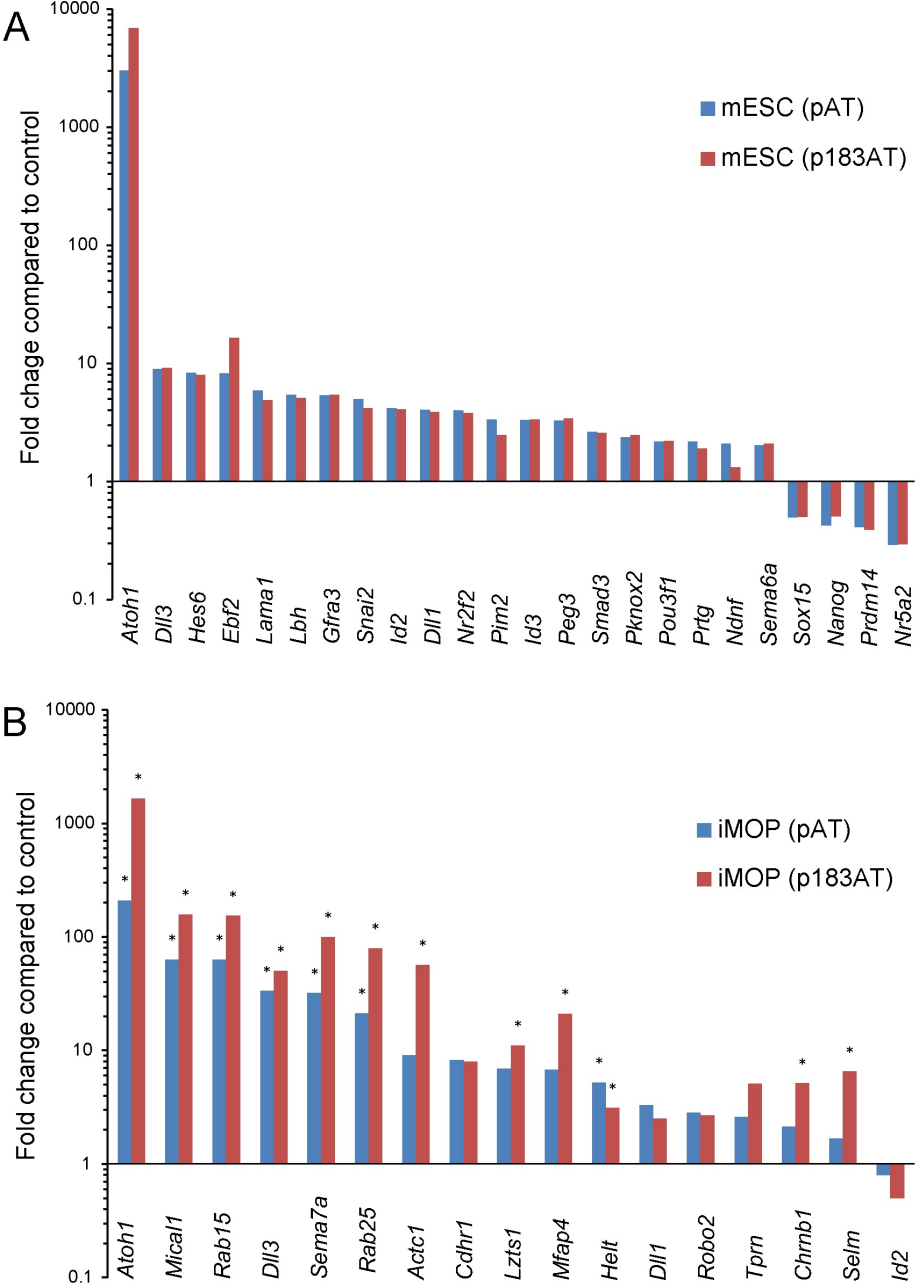
qRT-PCR validation of a subset of differentially expressed genes in mESC and iMOP cells. Graphs show linear fold change of differentially expressed genes by Taqman qPCR detection in Atoh1-expressing cells (blue) and Atoh1/miR-183 family-expressing cells (red) compared to control in mESCs (A) and iMOP cells (B). Data were normalized to detection of 18S ribosomal RNA. All values represent statistically significant differences for mESCs except for Ndnf expression in Atoh1/miR-183 family-expressing cells, and statistically significant differences are indicated by asterisks for iMOP cells (n = 3, Student’s t-test p<0.05).

To validate differential gene expression detected by microarray analyses, Taqman qRT-PCR was performed comparing gene expression for different conditions to control. In both Atoh1*-* and Atoh1/miR-183 family-expressing mESCs, upregulation was verified for *Atoh1*, *Dll3*, *Dll1*, *Snai2*, *Hes6*, *Lbh*, *Id2*, *Id3*, *Ebf2*, *Prtg*, *Pknox2*, *Nr2f2*, *Pou3f1*, *Smad3*, *lama1*, *Peg3*, *Pim2*, *Car2*, *Gfra3* and *Sema6a*, and downregulation was verified for *Nanog*, *Sox15*, *Prdm14* and *Nr5a2* (n = 3, Student’s t-test p<0.05) (Fig 9A). In both Atoh1*-* and Atoh1/miR-183 family-expressing iMOP cells, upregulation was validated for *Atoh1*, *Dll3*, *Helt*, *Mical1*, *Rab15*, *Rab25* and *Sema7a*, and Atoh1/miR-183 family-expressing cells additionally validated upregulation of *Actc1*, *Lzts1*, *Mfap4*, *Chrnb1*, and *Selm* (n = 3, Student’s t-test p<0.05) (Fig 9B).

## Discussion

Our work describes how different combinations of Atoh1 and miR-183 family expression promote HC fate in pluripotent and multipotent cells. Global gene expression analysis provided an unbiased and thorough approach to examine the influence of these factors. In addition, utilization of two different developmental cell culture models facilitated understanding of the contextual prerequisite for Atoh1 and miR-183 family roles during development. We used mESCs as a model of pluripotent stem cells, which have been commonly proposed in approaches for cell replacement therapies, and iMOP cells as a model of otic fate-restricted yet multipotent self-renewing cells capable of terminal differentiation upon removal of bFGF [20].

Results generally show that as more factors are introduced in each cell culture model, an increase in the number of differentially expressed genes is observed. The percentage of upregulated genes also identified as enriched in cochlear HCs [13] can be considered to reflect the efficiency of reprograming toward a HC fate. Atoh1-expressing mESCs exhibited approximately 20% with no substantial difference between Atoh1 alone or in combination with the miR-183 family. By comparison, iMOP cells showed greater percentages of upregulated genes identified as enriched in HCs, where Atoh1 expression alone yielded 28% and the combination of Atoh1/miR-183 family yielded 38%. Our data indicate that iMOP cells are more receptive to HC programming, and that the miR-183 family works synergistically with Atoh1 in this cell culture model.

Comparison of the number of differentially upregulated genes with Atoh1 expression in each cell culture model showed that twice as many genes were upregulated in mESCs than in iMOP cells. One possible explanation is the presumed epigenetic difference between the two cell types, where there is a large amount of transcriptionally active chromatin in mESCs compared to differentiated lineages [27]. Therefore, more Atoh1 target genes might be transcriptionally accessible in mESCs than in iMOP cells.

Enriched gene ontological categories in Atoh1-expressing mESCs revealed a large number of developmental proteins and transcriptional regulators, some of which are involved in epithelial cell differentiation. These categories are less represented in Atoh1-expressing iMOP cells, which showed enrichment in differentiation and protein localization categories. Since iMOP cells are already specified to an epithelial fate, the impact of Atoh1 appears directed toward inducing terminal differentiation. In both cell culture models, Atoh1 expression upregulated multiple genes involved in neurogenesis. A recent study in which Atoh1 was expressed in embryonic stem cells did not induce cells toward a HC fate; cells were instead driven toward neuronal differentiation [28]. Our results are consistent with these findings and provide further evidence of the role of Atoh1 in neurogenesis when used as a sole factor for pluripotent stem cell programming. The striking difference between mESCs and iMOP cells with regard to upregulated genes and their functional categories upon Atoh1 expression confirms the previous claim that the cellular context in which Atoh1 functions determines its selection of target genes [29].

Our analysis showed few Atoh1-regulated genes that are contextually independent including the Notch ligand-encoding genes *Dll1* and *Dll3*. Previous studies showed that mouse HCs express *Dll1* and *Dll3* around E14.5-E15.5 [30,31], and that the Notch signaling pathway plays a key role in determining HC and supporting cell fates through a lateral inhibition receptor/ligand interaction [32,33]. Atoh1 has also been shown to regulate Notch ligand genes and thereby directs the differentiation of HCs [34], cerebellar granule cells [35] and intestinal secretory cells [36]. Our data show that the inductive effect of Atoh1 on Delta ligands is contextually independent in our cell culture models and thereby provide further evidence for this consistent role of Atoh1 in different cellular contexts.

Interestingly, *Id2* gene expression was upregulated in mESCs and downregulated in iMOP cells upon Atoh1 expression. ID proteins function as negative regulators of basic helix-loop-helix proteins through the formation of inactive heterodimers [37]. It was previously shown that the expression of ID proteins and Atoh1 overlapped in cochlear progenitor cells before cellular differentiation, but a specific downregulation of ID protein expression was observed in individual cells that differentiated as HCs [38]. Our results indicate that Atoh1 is capable of inducing two opposite effects on *Id2* expression depending on transcriptional context. Upon examining contextual differences between unmanipulated mESCs and iMOP cells, we found 14-fold upregulation of *Bmp4* in mESCs compared to iMOPs. Previous studies have shown that ID proteins are potently induced by bone morphogenic proteins (BMPs) in different cell types [39–41], and BMP4 in particular is an inducer of *Id2* gene expression [42]. Based on these observations, it appears that BMP4 is one of the contextual modulators of Atoh1 function.

Expression of the miR-183 family alone was not sufficient to drive the expression of HC-specific genes in either cell culture model, but the combination of miR-183 family and Atoh1 showed a substantial increase in the number of upregulated HC genes in both cell models. This aligns well with previous evidence that miRNAs fine tune gene expression to reinforce a specific cell fate during development [43].

Among the miR-183 family predicted target genes within the list of downregulated genes in miR-183 family-expressing iMOP cells are sprouty homolog proteins. *Spry4* is an inhibitor of the receptor-transduced mitogen-activated protein kinase (MAPK) signaling pathway and is shown to be expressed during Zebrafish otic vesicle development [44]. Furthermore, overexpression of *Spry4* caused a reduction in the size of otic vesicles. *Spry4* expression during mouse inner ear development shows more than 17-fold downregulation in cochlear HCs versus non-HCs between E16 and P0, and this downregulation is maintained until at least P7 [26]. By repressing *Spry4* gene expression, the miR-183 family may reduce MAPK signaling inhibition and thereby potentially affect multiple cellular processes including proliferation and survival. A recent study showed that in human embryonic stem cells, *Spry4* knockdown enhanced cell survival [45]. Repression of Sprouty protein expression might be one possible way through which the miR-183 family supports HC survival during development and differentiation.

Our data revealed *Tbx1* as another predicted target gene that is downregulated in iMOP cells expressing the miR-183 family. *Tbx1* is a T-box containing transcription factor that is expressed early in otocyst development, and loss-of-function obstructs inner ear development at an early otocyst stage [46]. Additionally, transgenic mice overexpressing *Tbx1* exhibit sensorineural hearing loss and malformation of the cochlear duct [47]. Both gain-of-function and loss-of-function studies indicate that the inner ear is highly sensitive to *Tbx1* expression levels and underscore the importance of precise *Tbx1* regulation. Our data suggest that the miR-183 family is involved in modulating the expression of *Tbx1* to facilitate proper HC differentiation. This role for the miR-183 family is supported by a previous study in which overexpression of *miR-182* was shown to inhibit the expression of *Tbx1* in the cultured otic progenitor/stem cells [48].

Combined expression of Atoh1 and miR-183 family in iMOP cells was sufficient to specifically upregulate some HC enriched genes including *Nhlh1*, *Chrnb1* and *Myo6* that were not upregulated in Atoh1-expressing cells. *Nhlh1* is a bHLH transcription factor that is expressed at E18.5 in all sensory regions of the inner ear including inner and outer HCs of the cochlea [49]. This timing directly follows the expression of Atoh1 and the miR-183 family [50]. Two weeks after birth, *Nhlh1* expression decreases significantly in HCs and is undetectable by P30 [49], suggesting that *Nhlh1* is primarily involved in perinatal development of the inner ear. The expression of *Nhlh1* genes was also evident in other sensory structures during development such as the ganglion cell layer of the retinal epithelium at E14.5, and the olfactory system [51]. The expression dynamics of *Nhlh1* correlate well with miR-183 family member expression suggesting a possible role for the miR-183 family in *Nhlh1* induction.

Some genes showed upregulation in Atoh1-expressing iMOP cells, but no change with combined Atoh1/miR-183 family expression, including *Helt*. *Helt* belongs to the bHLH-O family of transcriptional repressors and has been shown to regulate neuronal differentiation and specifically promote GABAergic neuronal fate [52]. This finding suggests a role for the miR-183 family in repressing Atoh1-induced effects that are not HC specific, thereby contextually reinforcing the HC fate.

Multiple genes previously demonstrated to lie downstream of Atoh1 did not show increased expression in Atoh1-expressing mESCs or iMOP cells. For example, transcription factor Barhl1 is expressed in hair cells around E14.5 and relies on Atoh1 as an upstream regulator [53], but it was not upregulated in either cell culture model. Similarly, transcription factor Gfi1 is expressed in hair cells around E15.5 [54] and functions downstream of Atoh1 [55], and transcription factor Pou4f3 is directly regulated by Atoh1 [56], but neither is upregulated in Atoh1-expressing mESCs or iMOP cells. A likely explanation is that mESCs and iMOP cells lack other essential factors that work in concert with Atoh1 to activate these target genes. Alternatively, the duration or level of expression of Atoh1 might be insufficient to induce such target genes.

Although our data show multiple novel downstream effects for Atoh1 and the miR-183 family, further studies are required to distinguish direct versus indirect gene targets for each factor. In conclusion, our analyses show that the role of Atoh1 in development is contextually dependent and is capable of regulating HC genes more efficiently in a later developmental context. Our study also demonstrates for the first time that the combination of Atoh1 and miR-183 family is more efficient than Atoh1 alone in driving the expression of HC genes. Results from this study and further analysis of target genes and pathways could ultimately lead to therapeutic treatments to protect HCs and prevent hearing loss.

## Supporting information

S1 Table Microarray data and differential expression analysis for mESCs transfected with different expression vector combinations. (XLSX)

S2 Table Microarray data and differential expression analysis for iMOP cells transfected with different expression vector combinations. (XLSX)

S3 Table Taqman assay IDs for qRT-PCR validation of differentially expressed genes in transfected mESCs and iMOP cells. (DOCX)
