# Supplementary material for: Transcriptome-wide comparison of the impact of Atoh1 and miR-183 family on pluripotent stem cells and multipotent otic progenitor cells
Source: PLoS One. 2017 Jul 7;12(7):e0180855. doi: 10.1371/journal.pone.0180855 (PMC5501616; doi:10.1371/journal.pone.0180855)
Supplement: S3 Table — (DOCX) [file pone.0180855.s003.docx]

**S3 Table**. Taqman assays for quantitative RT-PCR

| **Gene Symbol** | **Gene name** | **Taqman Assay ID** | **NCBI Gene Reference** |
| --- | --- | --- | --- |
| ***18S*** | Eukaryotic 18S rrna | Hs99999901_s1 | X03205.1 |
| ***Actc1*** | Actin, alpha, cardiac muscle 1 | Mm01333821_m1 | NM_009608.3 |
| ***Atoh1*** | Atonal homolog 1 (Drosophila) | Mm00476035_s1 | NM_007500.4 |
| ***Cdhr1*** | Cadherin-related family member 1 | Mm00499982_m1 | NM_130878.2 |
| ***Chrnb1*** | Cholinergic receptor, nicotinic, beta polypeptide 1 (muscle) | Mm00680412_m1 | NM_009601.4 |
| ***Dll1*** | Delta-like 1 (Drosophila) | Mm01279269_m1 | NM_007865.3 |
| ***Dll3*** | Delta-like 3 (Drosophila) | Mm00432854_m1 | NM_007866.2 |
| ***Ebf2*** | Early B cell factor 2 | Mm00438625_m1 | NM_010095.6 |
| ***Gfra3*** | Glial cell line derived neurotrophic factor family receptor alpha 3 | Mm00494589_m1 | NM_010280.3 |
| ***Helt*** | Helt bhlh transcription factor | Mm00723333_g1 | NM_173789.4 |
| ***Hes6*** | Hairy and enhancer of split 6 | Mm00517097_g1 | NM_019479.3 |
| ***Id2*** | Inhibitor of DNA binding 2 | Mm00711781_m1 | NM_010496.3 |
| ***Id3*** | Inhibitor of DNA binding 3 | Mm01188138_g1 | NM_008321.2 |
| ***Lama1*** | Laminin, alpha 1 | Mm01226102_m1 | NM_008480.2 |
| ***Lbh*** | Limb-bud and heart | Mm00522506_m1 | NM_029999.4 |
| ***Lzts1*** | Leucine zipper, putative tumor suppressor 1 | Mm01345507_m1 | NM_199364.2 |
| ***Mfap4*** | Microfibrillar-associated protein 4 | Mm00840681_m1 | NM_029568.2 |
| ***Mical1*** | Microtubule associated monooxygenase, calponin and LIM domain containing 1 | Mm00506780_m1 | NM_138315.2 |
| ***Nanog*** | Nanog homeobox | Mm02384862_g1 | NM_028016.2 |
| ***Ndnf*** | Neuron-derived neurotrophic factor | Mm00549567_m1 | NM_172399.3 |
| ***Nr2f2*** | Nuclear receptor subfamily 2, group F, member 2 | Mm00772789_m1 | NM_009697.3 |
| ***Nr5a2*** | Nuclear receptor subfamily 5, group A, member 2 | Mm01285336_m1 | NM_030676.3 |
| ***Peg3*** | Paternally expressed 3 | Mm01337379_m1 | NM_008817.2 |
| ***Pim2*** | Proviral integration site 2 | Mm00454579_m1 | NM_138606.2 |
| ***Pknox2*** | Pbx/knotted 1 homeobox 2 | Mm00455847_m1 | NM_148950.3 |
| ***Pou3f1*** | POU domain, class 3, transcription factor 1 | Mm00843534_s1 | NM_011141.2 |
| ***Prdm14*** | PR domain containing 14 | Mm01237814_m1 | NM_001081209.2 |
| ***Prtg*** | Protogenin homolog (Gallus gallus) | Mm00615501_m1 | NM_175485.4 |
| ***Rab15*** | RAB15, member RAS oncogene family | Mm00513627_m1 | NM_134050.4 |
| ***Rab25*** | RAB25, member RAS oncogene family | Mm00444175_m1 | NM_016899.4 |
| ***Robo2*** | Roundabout homolog 2 (Drosophila) | Mm00620713_m1 | NM_175549.4 |
| ***Selm*** | Selenoprotein M | Mm00459806_m1 | NM_053267.2 |
| ***Sema6a*** | Sema domain, transmembrane domain (TM), and cytoplasmic domain, (semaphorin) 6A | Mm00444441_m1 | NM_018744.2 |
| ***Sema7a*** | Sema domain, immunoglobulin domain (Ig), and GPI membrane anchor, (semaphorin) 7A | Mm00441361_m1 | NM_011352.2 |
| ***Smad3*** | SMAD family member 3 | Mm01170760_m1 | NM_016769.4 |
| ***Snai2*** | Snail homolog 2 (Drosophila) | Mm00441531_m1 | NM_011415.2 |
| ***Sox15*** | SRY (sex determining region Y)-box 15 | Mm00839542_g1 | NM_009235.2 |
